# Temporal patterns of hospitalizations for diabetic ketoacidosis in children and adolescents

**DOI:** 10.1371/journal.pone.0245012

**Published:** 2021-01-07

**Authors:** Arpita Kalla Vyas, Lavi Oud

**Affiliations:** 1 Department of Pediatrics, College of Medicine, California Northstate University, Elk Grove, California, United States of America; 2 Division of Pulmonary and Critical Care Medicine, Department of Internal Medicine, Texas Tech University Health Sciences Center at the Permian Basin, Odessa, Texas, United States of America; Brown University Warren Alpert Medical School, UNITED STATES

## Abstract

**Objectives:**

To examine the temporal patterns of hospitalizations with diabetic ketoacidosis (DKA) in the pediatric population and their associated fiscal impact.

**Methods:**

The Texas Inpatient Public Use Data File was used to identify hospitalizations of state residents aged 1month-19 years with a primary diagnosis of DKA during 2005–2014. Temporal changes of population-adjusted hospitalization rates and hospitalization volumes were examined for the whole cohort and on stratified analyses of sociodemographic attributes. Changes in the aggregate and per-hospitalization charges were assessed overall and on stratified analyses.

**Results:**

There were 24,072 DKA hospitalizations during the study period. The population-adjusted hospitalization rate for the whole cohort increased from 31.3 to 35.9 per 100,000 between 2005–2006 and 2013–2014. Hospitalization volume increased by 30.2% over the same period, driven mainly by males, ethnic minorities, those with Medicaid insurance and uninsured patients. The aggregate hospital charges increased from approximately $69 million to $130 million between 2005–2006 and 2013–2014, with 66% of the rise being due to increased per-hospitalization charges.

**Conclusions:**

There was progressive rise in pediatric DKA hospitalizations over the last decade, with concurrent near-doubling of the associated fiscal footprint. Marked disparities were noted in the increasing hospitalization burden of DKA, born predominantly by racial and ethnic minorities, as well as by the underinsured and the uninsured. Further studies are needed to identify scalable preventive measures to achieve an equitable reduction of pediatric DKA events.

## Introduction

The incidence of pediatric diabetes is increasing in the United States (US) [1]. However, despite its rising population burden, an earlier report documented no concomitant rise in the population-adjusted rate of diabetes-associated hospitalizations [2].

Although no recent data were reported, to our knowledge, on pediatric diabetes associated hospitalizations in the US, a report by Witt and colleagues documented nearly 28% reduction in the rate of pediatric hospitalizations with a Major Diagnostic Category (MDC) of Endocrine, nutritional and metabolic diseases between 2000 and 2012 [3]. This later downtrend was part of a broader reduction in hospitalization rates for most MDCs [3] and a documented decrease in both volumes and rates of pediatric hospitalizations in the US [4]. These latter trends suggest marked improvement in outpatient preventive efforts that may have reduced the need for inpatient care in the pediatric population.

Diabetes ketoacidosis (DKA) remains the major acute complication of pediatric diabetes, but is considered largely a preventable one [5,6]. Improving care has transformed pediatric DKA into a relatively low-severity complication at a population level in the US, when considered based on its average short hospital stay [7] and very low hospital mortality [8]. Institute-specific guidelines for the management of DKA, derived from the consensus statements for DKA management, including those from American Diabetes Association [9] International Society of Pediatric Adolescent Diabetes [10] and the European Society of Paediatric Endocrinology and Pediatric Endocrine Society [11], are applied across the country and are in part responsible for the improved care. However, there exist variability is the management of the condition [12] and hospitalized patients are frequently admitted to ICU [13], with its attendant risks [14,15] and, although uncommon, can have life-threatening complications [8,16]. In addition, the health-related [17] and economic tolls [18] of DKA extend beyond hospital discharge.

In contrast with the available data on the overall diabetes-associated hospitalization, only limited population-level data were reported, to our knowledge, on the temporal trajectories of the hospitalization burden of pediatric DKA and its fiscal impact. A recent study by Patel and colleagues estimated that there were nearly 250,000 hospitalizations with pediatric DKA in the US during 2002–2012 [8]. A more recent report by Desai et al showed an increasing hospitalization volume of DKA in the US among those aged 0–17 years between 2003 and 2014 [19]. However, the authors did not provide data on the corresponding population-adjusted hospitalization rates [8,19], nor on the details of the hospitalization burden across the demographic strata in the pediatric population [19]. It is thus presently unclear whether the burden of DKA hospitalizations, adjusted for population growth, has followed a stable [2] or possibly decreasing [3] hospitalization burden of pediatric diabetes. The national annual cost of pediatric DKA hospitalizations has been estimated at $90 million [20]. However, the contemporary temporal patterns of the fiscal impact of DKA hospitalizations are unknown. Contemporary data on the temporal patterns of pediatric DKA hospitalizations can inform health policy and future studies to improve our understanding of the factors driving the observed hospitalization patterns and to provide means for practice improvement to reduce DKA-related hospitalizations.

We conducted a population-level study of the burden of pediatric DKA hospitalizations to examine a) population-adjusted temporal trends of hospitalization rates, both overall and demographically-stratified b) temporal changes in total and demographically-stratified hospitalization volumes and their associated fiscal footprint.

## Material and methods

This was a retrospective, population-based cohort study. We used a publicly available, de-identified data set, and thus this study was determined to be exempt from formal review by the Texas Tech Health Sciences Center’s Institutional Review Board.

### Data sources and study population

We used the Texas Inpatient Public Use Data File (TIPUDF), an administrative data set maintained by the Texas Department of State Health Services [21] that captures approximately 97% of all hospitalizations in the state. The use of TIPUDF has been previously described [22]. Hospitalizations with DKA as primary diagnosis were identified among state residents, aged 1 month-19 years, during the years 2005–2014, using International Classification of Diseases, Ninth Revision (ICD-9), Clinical Modification codes 250.10–250.13. Prior report has demonstrated that ICD-9 codes cannot distinguish accurately between type 1 and type 2 diabetes in children and adolescents [23]. Hence, we combined both diabetes types in our analyses, similarly to the approach used by other investigators who used administrative data sets to examine hospitalization patterns associated with diabetes [2] and specifically with DKA [7,19]. Because TIPUDF provides discharge-level, rather than patient-level information, precluding accounting for repeated admissions in the data set, we report the number of hospitalizations as the unit of analysis, rather than the number of patients. We excluded hospitalizations with diagnoses of HIV infection, alcohol or substance abuse-related disorders because their age and gender data are suppressed by the state. US census data were used to obtain general population data for the state of Texas.

### Outcomes

The primary outcome was the temporal pattern of DKA hospitalizations. The secondary outcome was the temporal change in the aggregate and per-hospitalization charges.

The patterns of DKA hospitalizations were examined in two ways. First, we examined the temporal trends of population-adjusted hospitalization rates for the whole cohort and within demographic strata. In addition, we examined changes in the volume of hospitalizations during study period. This second approach provides a complementary representation of the hospitalization burden of DKA, allowing more direct examination of the relative contributions of patients’ demographic attributes to hospitalization volume within a specific timeframe and over time.

### Study variables

We extracted information on the age, gender, race/ethnicity, health insurance, and hospital charges. In addition, we calculated the Deyo modification of the Charlson Comorbidity index [24] and the number of organ failures, as reported by Martin and colleagues [25].

Hospital charges were adjusted for inflation using the consumer price index and are reported as 2014 US dollars [26]. TIPUDF and the state of Texas do not provide tools for conversion of hospital charges to costs.

### Data analysis

We summarized categorical variables as numbers and percentages and continuous variables as means and standard deviation (SD). Chi-square test and the Mann-Whitney test were used for group comparisons involving categorical and continuous variables, respectively.

The annual population-adjusted hospitalization rates were estimated by dividing the number of hospitalizations by the denominator of state population for the whole cohort and for the age, gender, and race/ethnicity groups. We used 2-year moving averages of hospitalization rates to reduce year-to-year fluctuations [1] and expressed findings per 100,000 population. We used log-transformed least squares regression to model the temporal trends of annual hospitalizations rates as dependent variable for the whole cohort and within the abovementioned demographic strata. The results were expressed as average annual percent change (AAPC) and its 95% confidence interval (95% CI).

Changes in the volume of DKA hospitalizations during the study period were examined using 2-year data grouping to improve the robustness of findings. Hospitalization volumes were compared for the whole cohort and within the age, gender, race/ethnicity, and health insurance strata.

We examined changes over time in the economic burden of DKA hospitalizations as aggregate and per-hospitalization charges, using 2-year grouped data as described above, for the whole cohort and within the demographic strata described for the volume of hospitalizations.

Temporal changes in the aggregate hospital charges can reflect simply changes in the number of hospitalizations, but also changes in per-hospitalization charges. In order to refine the interpretation of the changing fiscal footprint of DKA hospitalizations we further examined the relative contribution of changes in the per-hospitalization charges to the increase in aggregate charges for the whole cohort and on stratified analyses for demographic characteristics.

Increases in per-hospitalization charges can be affected in turn by factors unrelated to patient-specific attributes (e.g., hospitals’ business practices) as well as by increases in care intensity, with the later driven largely by patients’ complexity. We examined whether there were changes over time in the Deyo comorbidity index and the number of organ failures, representing patients’ burden of chronic illness and severity of illness, respectively, using both as proxy measures for patient’s complexity of illness for the whole cohort and within the abovementioned demographic strata. In order to examine whether the Deyo comorbidity index and the number of organ failures predict hospital charges, linear regression analyses were carried out for each, showing that hospital charges for our cohort rose (coefficient [standard error]; p value) by $3,843 [341] (p <0.0001) per 1 point of the Deyo comorbidity index and by $10,492 [357] (<0.0001) per each 1 organ failure.

Data management was performed using Excel and Access (Microsoft, Redmond, Washington) and statistical analyses were performed with Prism V7 (Graphpad Software, Inc., San Diego, USA) and MedCalc version 18 (MedCalc Software, Ostend, Belgium). A 2-sided *p* value < 0.05 was considered statistically significant.

## Results

From 2005 through 2014 there were 24,072 hospitalizations with DKA. The characteristics of DKA hospitalizations are outlined in Table 1. Most DKA hospitalizations were female (54.3%), 54.3% were non-white, and 11.6% lacked health insurance. Only a small minority (538 [2.2%]) of DKA hospitalizations were in rural hospitals, while the remainder were managed in metropolitan facilities, and 10,985 (45.5%) were admitted to teaching hospitals.

**Table 1:** The characteristics of hospitalizations with diabetic ketoacidosis in Texas, 2005–2014.

Category	Hospitalizations^a^
**All**	24,072
**Age (years)**	
1 month-<5	1,291 (5.4)
5-<10	2,983 (12.4)
10-<15	7,711 (32.0)
15–19	12,087 (50.2)
**Gender**	
Male	11,000 (45.7)
Female	13,072 (54.3)
**Race/ethnicity**^**b**^	
White	10,994 (45.7)
Hispanic	6,908 (28.7)
Black	4,538 (18.9)
Other	1,616 (6.7)
**Health insurance**^**c**^	
Private	11,412 (47.4)
Medicaid	9,105 (37.8)
Self-pay	2,796 (11.6)
Other	747 (3.1)
**Deyo comorbidity index**^**d**^	0.11 (0.38)
**Number of organ failures**^**d**^	0.10 (0.36)
**Admitting Facilities**	
Rural	538(2.2)
Teaching	10,985 (45.5)

^a^ Hospitalizations are expressed as number (%), with the exception of the Deyo comorbidity index and the number of organ failures.

^b^ Race/ethnicity data were missing in 16 (<0.1%) hospitalizations.

^c^ Health insurance data were missing in 12 (<0.1%) hospitalizations.

^d^ Expressed as mean (standard deviation).

### Temporal trends in rates of DKA hospitalizations

The temporal trends of population-adjusted rates of DKA hospitalization for the whole cohort and the demographic strata are detailed in Table 2. The data on the overall and stratified annual number of hospitalizations with DKA and the corresponding Texas population are detailed in S2 Table in S1 File. The DKA hospitalization rate for the whole cohort rose from 31.3 to 35.9 per 100,000 population from 2006 to 2014. The rates of DKA hospitalizations increased with age, being over 7-fold higher among those aged 15–19 years, as compared to those aged 1 month—< 5 years. The rate of DKA hospitalizations was consistently higher among females, and has been nearly 3-fold higher among blacks than among Hispanics.

**Table 2:** Temporal trends of population-adjusted rates of hospitalization with diabetic ketoacidosis in Texas^a^.

	2006	2007	2008	2009	2010	2011	2012	2013	2014	AAPC (95% CI)^b^	p value
			Hospitalizations/100,000 population/year								
**All**	31.3	33	31.5	32.2	33.6	35.1	36.9	36	35.9	2.2 (0.91 to 3.4)	0.0040
**Age (years)**											
1 month-<5	7.7	7.6	7.4	7.3	7.4	8.9	10	9.7	9.7	3.9 (1.5 to 6.2)	0.006
5-<10	18.5	18.1	16.3	15.4	14.5	15	15.9	15.8	16.6	-1.2 (-3.4 to 1.0)	0.2600
10-<15	38	41.1	39	40.4	43.9	44.7	46.2	44.8	41.7	1.7 (-0.3 to 3.7)	0.0930
15–19	56.2	60.1	58.6	61	64.3	67.2	70.2	68	70.1	3.1(1.9 to 4.4)	0.0010
**Gender**											
Male	26.8	29.6	28.1	27.9	29.9	31.9	33.4	32.3	32.8	2.9 (1.3 to 4.6)	0.0040
Female	36	36.6	35.1	36.7	37.5	38.5	40.5	39.7	39.3	1.5(0.4 to 2.7)	0.0130
**Race/ethnicity**											
White	37.9	42	40	40.2	41.8	44.6	48.5	47.7	48.4	3.3 (1.5 to 5.2)	0.0030
Hispanic	18	19	18.9	19.7	21.8	22.3	22.3	22.4	22.4	3.3 (1.4 to 5.3)	0.0050
Black	48.3	47.4	44.4	47.6	53.1	57.4	56.6	54	59.3	3.0 (1.9 to 4.2)	<0.0001
Other	59.9	58	53.8	52.5	39.1	39.1	50.1	44	33.6	-6.1 (-10.6 to -1.6)	0.0100

^a^ Rates are based on the number of hospitalizations in the given year and the preceding year (2-year moving average).

^b^ AAPC (95% CI) indicates average annual percent change (95% confidence interval).

Hospitalizations rates rose fastest among those aged 1 month-< 5 years (AAPC 3.9%/year), while no change was noted among those aged 5-<15 years. The pace of rising DKA hospitalization rate was nearly 2-fold higher among males than females (2.9%/year vs. 1.5%/year, respectively), while the annual change in hospitalization rates was comparable among whites, blacks and Hispanics.

### Changes in the volume of DKA hospitalizations

The changes in the volume of DKA hospitalizations during the study period are described in Table 3. The hospitalization volume rose 30.2% from 2005–2006 to 2013–2014. The highest change in hospitalization volume was among those aged 15–19 years (+38%), while the lowest change was among the 5-<10 year group (+7.4%).

**Table 3:** Changes in the volume of hospitalizations with diabetic ketoacidosis in Texas.

Category	2005–2006	2013–2014	Change from 2005–2006 to 2013–2014 (%)	*p* value^a^
**All**	4,153	5406	30.2	
**Age (years)**				0.001
1 month-<5	226 (5.4)^b^	301 (5.6)	33.2	
5-<10	608 (14.6)	653 (12.1)	7.4	
10-<15	1316 (31.7)	1688 (31.2)	28.3	
15–19	2003 (48.2)	2764 (51.1)	38	
**Gender**				0.0043
Male	1818 (43.8)	2525 (46.7)	38.9	
Female	2335 (56.2)	2881 (53.3)	23.4	
**Race/ethnicity**				< 0.0001
White	2003 (48.2)	2374 (43.9)	18.5	
Hispanic	1047 (25.2)	1655 (30.6)	58.1	
Black	818 (19.7)	1038 (19.2)	26.9	
Other	281 (6.8)	333 (6.2)	18.5	
**Health insurance**				< 0.0001
Private	2084 (50.2)	2410 (44.6)	15.6	
Medicaid	1503 (36.2)	2203 (40.8)	46.6	
Self-pay	436 (10.5)	645 (11.9)	47.9	
Other	128 (3.1)	147 (2.7)	14.8	

^a^ Chi-square test of the change in the volume of hospitalizations with diabetic ketoacidosis within individual strata.

^b^ parenthesized figures represent the percent of the volume of hospitalization within a specific stratum out of the total hospital volume for the examined year period (example: Hospitalizations with diabetic ketoacidosis aged 1 month—<5 years in 2005–2006 represent 5.4% of all hospitalizations with diabetic ketoacidosis in these years).

Although the volume of DKA hospitalizations remained lower among males, their change in hospital volume was higher than that among females (38.9% vs. 23.4%, respectively) and the former accounted for 56.4% of the increase in hospitalization volume during the study period.

Minority DKA hospitalizations accounted for 70.4% of the increase in hospitalization volume and the highest change in hospitalization volume was among Hispanics (58.1%).

DKA hospitalizations lacking health insurance and those with Medicaid had the highest change in hospitalization volume (47.9% and 46.6%, respectively), a change nearly 3-fold higher than among those with private insurance.

### Changes in hospital charges among DKA hospitalizations

The changes in the aggregate and per-hospitalization charges of DKA hospitalizations for the whole cohort and the demographic groups are detailed in Table 4. The aggregate charges for DKA hospitalizations rose 89% from approximately $69 million to $130 million from 2005–2006 to 2013–2014, respectively.

**Table 4:** Changes in the aggregate and per-hospitalization charges among hospitalizations with diabetic ketoacidosis in Texas.

Category	Aggregate hospital charges^a^					Per-hospitalization charges^a^		
	2005–2006 (n = 4,153)	2013–2014 (n = 5,406)	Change from 2005–2006 to 2013–2014 (%)	Change from 2005–2006 to 2013–2014 due to per-hospitalization charges (%)^b^	*p* value^c^	2005–2006	2013–2014	Change from 2005–2006 to 2013–2014 (%)
**All hospitalizations**	68,582,193	129,645,720	89^d^	66		16,513	23,982	45
**Age (years)**					< 0.0001			
1 month-<5	4,322,510 (6.3)^e^^,^ ^f^	9,045,325 (7.0)	109	70		19,126	30,051	57
5-<10	9,801,298 (14.3)	15,867,807 (12.2)	62	88		16,120	24,300	51
10-<15	21,271,593 (31.0)	41,254,164 (31.8)	94	70		16,163	24,440	51
15–19	33,186,792 (48.4)	63,478,424 (49.0)	91	58		16,568	22,966	39
**Gender**					< 0.0001			
Female	39,538,158 (57.7)	70,888,610 (54.7)	79	70		16,933	24,606	45
Male	29,044,035(42.3)	58,757,110 (45.3)	102	62		15,975	23,270	46
**Race/ethnicity**					< 0.0001			
White	29,618,253 (43.2)	54,833,251 (42.2)	85	78		14,787	23,097	56
Hispanic	18,861,227 (27.5)	41,621,531 (32.1)	121	52		18,015	25,149	40
Black	14,982,412 (21.8)	25,328,255 (19.5)	69	61		18,315	24,401	33
Other	5,080,923 (7.4)	7,783,824 (6.0)	53	65		18,082	23,375	29
**Health insurance**					< 0.0001			
Private	33,787,648 (49.3)	54,584,861 (42.1)	62	75		16,212	22,649	40
Medicaid	26,529,538 (38.7)	55,629,133 (42.9)	110	58		17,651	25,252	43
Self-pay	6,200,738 (9.0)	16,132,409 (12.4)	160	70		14,222	25,011	76
Other	2,038,919 (3.0)	3,284,087 (2.5)	61	76		15,929	22,341	40

^a^ all charges are inflation-adjusted and expressed as 2014 US dollars.

^b^ the percent change is an expression of the proportion of the increase in the dollar amount of the aggregate hospital charges in 2013–2014 that cannot be accounted for by the expected rise due to the corresponding increase in the number of hospitalizations (if there was no change in the charge per-hospitalization); thus, as an interpretive example, only about a third of the 89% rise in the aggregate charges for all hospitalizations in 2013–2014 has been due to the increase in the number of DKA hospitalizations, while most of the rise (66%) was due to the 45% increase in charge per-hospitalization.

^c^ Chi-square test of the change in the aggregate hospital charges within individual strata.

^d^ percent figures are rounded.

^e^ parenthesized figures represent percent of aggregate hospital charges within a specific stratum out of the aggregate hospital charges for the examined year period.

^f^ percent figure may not add to 100 due to rounding.

There has been wide variation in the rates of change of the aggregate charges within the examined demographic strata, being highest among the youngest DKA hospitalizations (+109%), males (+102%), Hispanics (+121%), and those lacking health insurance (+160%).

Although the volume of DKA hospitalizations rose substantially for the whole cohort and within each of the examined demographic groups, it accounted for only a relatively small portion of the observed change in aggregate hospital charges. Rather, the rise in inflation-adjusted per-hospitalization charges drove for 66% of the change observed in aggregate hospital charges for the whole cohort and the majority of the change in aggregate hospital charges within each of the examined demographic strata. The impact of increasing per-hospitalization charges on the rise in aggregate hospital charges was highest among DKA hospitalizations aged 5-<10 years, females, whites, and those with private or “other” health insurance.

Per-hospitalization charges varied, as expected, within the demographic strata. The highest per-hospitalization charges in 2005–2006 were among the youngest age group, females, blacks, and those insured by Medicaid and, with the exception of blacks, remained so in 2013–2014.

Per-hospitalization changes for the whole cohort rose 45% during the study period, with the rise varying substantially within the demographic groups, being greatest among the youngest age group (+57%), whites (+56%), and those lacking health insurance (+76%).

The changes in the Deyo comorbidity index and the number of organ failures for the whole cohort and the demographic strata are outlined in S3 Table in S1 File, with both rising for the whole cohort. On stratified analyses the Deyo comorbidity index, the number of organ failures, or both, rose for each of the examined demographic groups, with the exception of DKA hospitalizations aged 1 month-<10 years, those of “other” race, and with “other” health insurance.

## Discussion

In this population-based study of DKA hospitalizations of children and adolescents, the population-adjusted hospitalization rates and hospital volumes rose considerably over the past decade, with the latter driven predominantly by DKA episodes among adolescents, males, minorities, and those lacking health insurance or insured by Medicaid. The aggregate hospital charges for DKA hospitalizations nearly doubled during the study period, with the rise driven largely by increased per-hospitalization charges.

### Relationship to previous studies

The contemporary temporal patterns of population-adjusted hospitalization rates of pediatric DKA were not previously reported, to our knowledge. However, our findings of progressively increased DKA hospitalization rates contrast prior reports of stable hospitalization rate of pediatric diabetes [2] and more recently decreasing rates of all pediatric hospitalizations [4] and specifically those with MDC of Endocrine, nutritional, and metabolic disease [3].

The observed overall differences in hospitalization rates with DKA across age groups, gender, and race/ethnicity are in line with prior reports showing poorer metabolic control among adolescents [27,28] and, specifically, adolescent females [29], and among blacks [30]. We also found divergent temporal trends across age strata, with rising hospitalization rates confined to the youngest and oldest examined age groups. Our finding is corroborated by recent studies that also reported bimodal age distribution for DKA in type 1 diabetics [31,32]. Thus the incidence of type 1 diabetes mellitus, reported to peak in young children and older age groups [33], likely contributed to the increased hospitalization rate of DKA in these age groups.

Our finding of the hospitalization rate among males rising at nearly double the rate among females extends an earlier report of the markedly faster growth in the incidence of type 1 diabetes mellitus among males in the pediatric population [1]. However, the factors underlying the observed increased DKA hospitalization rate for the whole cohort and the observed trajectories across the examined demographic strata in this study cannot be determined through administrative data. Because the administrative data in TIPUDF do not allow identification of individual patients, our findings quantify temporal trends in hospitalization-related burden of pediatric DKA, rather than its population level burden.

The hospitalization volume of pediatric DKA in the present study rose 3-fold higher than that reported by Desai and colleagues in a national cohort (30.2% vs 10.1% [19], respectively). However, the sources of the difference could not be determined with our study design. The stratified examination of the changes in hospitalization volumes during the study period has demonstrated evolving disparities in hospitalization burdens, especially across the gender, race/ethnicity and health insurance strata. Although females continued to account for the majority of hospitalizations by the end of the study period, similar to prior reports [8,34], the rise in hospitalization volume was driven mostly by male hospitalizations. Together with observed faster rise in the population-adjusted hospitalization rate among males, these findings suggest, that if the current trends continue, males will account for the majority of pediatric DKA hospitalization burden in Texas within a few years.

We found that the burden of DKA hospitalizations involved predominantly minority children and adolescents, and especially Hispanics. Minority children and adolescents also accounted for the majority of the change in volume of DKA hospitalizations. Our finding that the population-adjusted hospitalization rates for DKA among Hispanics was lowest among the examined racial and ethnic groups contrasts with their predominance in the observed change in hospitalization volume during the study period. This apparent contrast likely reflects the combination of the lower prevalence of type 1 diabetes mellitus among Hispanics, as compared with whites and black children and adolescents [35], affecting the former, and the rising internal growth of the Hispanic population, coupled with increased external migration to the state [36], driving the latter trend. Another contributing factor for the rising volume of DKA in Hispanic children likely is the rising incidence of type 2 Diabetes Mellitus amongst Hispanics youth [37,38]. In this regard, Klingensmith GJ and colleagues noted approximately 11% of pediatric patients with T2DM presented in DKA at diagnosis [37]. However, is not possible to discern from de-identified administrative data set whether DKA hospitalization were due to type 1 or type 2 Diabetes Mellitus.

DKA hospitalizations without private health insurance were predominant in our cohort, in line with prior reports of pediatric hospitalizations [39] and accounted for nearly three quarters of the rise in hospitalization volume over time. Of note, the rate of children and adolescents hospitalized with DKA while lacking health insurance in this study was over 3-fold higher than that reported in national data for general pediatric hospitalization (11.6% vs.3.5% [3], respectively). It is unclear whether the markedly higher rate of uninsured DKA hospitalizations in the present study reflects general in-state status or that related only to the pediatric diabetic population. Children and adolescents with public insurance or lacking health insurance are known to be at an increased risk of DKA [40,41]. However, it remains unclear whether the predominance of uninsured DKA hospitalizations and of those with Medicaid insurance in the rising hospitalization volume has been due to a) the rising number of diabetic children and adolescents with these categories of health insurance; b) increasing risk over time for hospitalization with DKA among those with Medicaid or without health insurance or c) some combination of both factors.

Because DKA among children and adolescents with established (e.g., prevalent) diabetes is considered largely preventable [5,6] data about the relative contribution of prevalent vs. incident diabetes (that is, diabetes diagnosed at the time of DKA) to DKA hospitalizations over time can inform health care policy and estimates of expected impact of future preventive efforts. However, to our knowledge, there have been no contemporary reports of population-level estimates of incident vs. prevalent diabetes among pediatric DKA hospitalizations. The administrative data used in the present study preclude direct distinction of DKA hospitalizations among those with incident vs. prevalent diabetes. However, the relative contribution of each to DKA hospitalizations may be approximated using available national estimates of the incidence of pediatric diabetes [1] for the years 2002–2012 and the rates of pediatric DKA at the time of diagnosis of type 1 and type 2 diabetes mellitus for the years 2002–2010 [42]. Applying the latter data to the Texas pediatric population and to our cohort (see Supplementary (S) data and S1 Table in S1 File for a detailed approach) through the year 2010 (e.g., the latest year with reported data on the rates of DKA at time of diagnosis of type 1 and type 2 diabetes) suggests that the increasing burden of DKA hospitalizations in Texas during the first 6 years of our study may have been driven mostly by patients with prevalent diabetes, accounting for over 70% of DKA hospitalizations in the state.

Our study demonstrates the substantial fiscal impact of the rising hospitalization burden of DKA, showing nearly doubling of the aggregate hospital charges by the end of the study period. We have quantified for the first time, to our knowledge, the relative contribution of the rising hospitalization volume vs. per-hospitalization charges of pediatric DKA hospitalizations, showing that the latter accounts for over two thirds of the change in aggregate charges. We could not quantify the relative contribution of hospitals’ business practices vs patient-specific factors to the 45% rise in per-hospitalization charges in our cohort. However, the documented rise in the burden of chronic illness and severity of illness suggests that per-hospitalization charges may have increased in part due to increasing complexity of DKA hospitalizations over time. Although the specific factors underlying the rise in per-hospitalization charges related to disease complexity cannot be determined from the administrative data, several potential drivers may be postulated.

At its most basic, increases in the proxy measures of disease complexity, while small, would have contributed to the rising per-hospitalization charges.

However, more complex cost drivers need to be considered, that were reflected in the proxy measures of the Deyo Comorbidity index and the number of organ failures among DKA hospitalizations in our cohort. Thus, an increase in per-hospitalization charges of DKA hospitalizations may have reflected degradation over time in patients’ access to health care in the state of Texas, the quality of primary and specialty diabetes and general medical care, and possibly changes in timely recognition of developing DKA prior to hospitalization.

In addition, we have to consider whether the rising per-hospitalization charges of DKA hospitalizations in our cohort reflect evolving changes in the phenotype of pediatric DKA in Texas (and possibly in other populations), independent of the aforementioned comorbidity and pre-hospital care considerations.

Given the increasing hospitalization burden of pediatric DKA demonstrated in our study, examination of the abovementioned postulated drivers of rising per-hospitalization charges in more granular data can guide both health policy and clinical practice.

### Study implications

Our findings of increasing burden of DKA hospitalizations underscore the need to identify scalable approaches to prevent DKA events among children and adolescents. Workable preventive measures are especially urgent given the increasing transformation of DKA hospitalizations in Texas to be predominated by minorities, known to have poorer metabolic control. Similarly, health policy solutions are needed to address barriers to adequate primary and specialty care among the publicly insured and the increasingly uninsured noted among pediatric DKA hospitalizations in the state.

### Study limitations

Our study has several important limitations, in addition to those noted earlier, related predominantly to its retrospective design and use of administrative data. First, use of administrative data may have led to misclassification of some of the DKA hospitalizations. However, similar approach was used in other epidemiological studies [7,30,43]. Second, we could not distinguish between single DKA events and recurrent ones. Third, the indicators of burden of chronic illness and severity of illness may have been insufficiently sensitive to fully capture the complexity of patients’ illness. Last, it is unknown whether our observations reflect DKA hospitalization patterns in other states or nationally.

## Conclusions

Our study demonstrates increasing DKA hospitalizations among children and adolescents in Texas across multiple sociodemographic groups, associated with nearly doubling hospitalization fiscal footprint. However, marked disparities were noted in the hospitalization burden, born increasingly by racial and ethnic minorities, as well as by the underinsured and those lacking health insurance. Further studies are required to determine the factors underlying the observed evolving patterns of DKA hospitalizations in order to identify scalable preventive measures to achieve equitable reduction of DKA events.

## Supporting information

S1 File (DOCX)
