# Supplementary material for: Temporal patterns of hospitalizations for diabetic ketoacidosis in children and adolescents
Source: PLoS One. 2021 Jan 7;16(1):e0245012. doi: 10.1371/journal.pone.0245012 (PMC7790255; doi:10.1371/journal.pone.0245012)
Supplement: S1 File — (DOCX) [file pone.0245012.s001.docx]

Supplementary Digital Content to:

**Temporal patterns of Hospitalizations for Diabetic Ketoacidosis in Children and Adolescents**

Arpita Kalla Vyas, MD, Lavi Oud, MD,

**Table of Contents:**

1. Methods

Approximation estimate of DKA hospitalizations associated with incident diabetes

Table S1

1. Results

Tables S2 and S3

1. References
2. **Materials and Methods**

Approximation estimate of DKA hospitalizations associated with incident diabetes

We hypothesized that prevalent diabetes underlies the majority of DKA hospitalizations over time in our cohort.

We approximated the fraction of DKA hospitalizations related to incident diabetes (that is, DKA at diagnosis of diabetes) in our cohort using the following approach:

1. The incidence of type 1 and type 2 diabetes was derived from the estimates reported by Mayer-Davis et al (1) for the years 2002-2012, using 2-year moving averages and using the 0-19 year age group for type 1 diabetes and the 10-19 year age group for type 2 diabetes.

We used similar 2-year moving average approach to estimate the number of children and adolescents with incident type 1 and type 2 diabetes in Texas, applying the incidence data from Mayer-Davis to the Texas population obtained from the US Census data (0-19 years for type 1 diabetes and 10-19 years for type 2 diabetes). Using the age range of 0-19 years (rather than the 1 month-19 years age range in our cohort) to derive the number of children and adolescents newly diagnosed with type 1 diabetes resulted in an overestimate of incident diabetes, as part of a conservative approach when considering the corresponding contribution of prevalent diabetes to DKA hospitalizations.

1. The prevalence of DKA at time of diabetes diagnosis was derived from the study by Dabalea et al (2) for the years 2002-2010. The authors reported the prevalence of DKA at time of diabetes diagnosis over 3 periods: 2002-2003, 2004-2005, and 2008-2010.

We applied these prevalence data to the derived approximation of the number of children and adolescents newly diagnosed with type 1 and type 2 diabetes (from #1 above) in Texas as follows: the data on the prevalence of DKA at time of diabetes diagnosis for the years 2004-2005 were applied to the years 2005-2006, and the prevalence data for the years 2008-2010 were applied to the years 2007-2008, 2008-2009 and 2009-2010 of Texas data of the approximated numbers of incident type 1 and type 2 diabetes mellitus. We thus obtained estimates of the number of DKA events in Texas at time of diagnosis of type 1 and type 2 diabetes. We assumed that all DKA events at the time of diabetes diagnosis required hospitalization, again as part of a conservative approach when considering the corresponding contribution of prevalent diabetes to DKA hospitalizations.

1. The combined estimates of the number of DKA events for type 1 and type 2 diabetes in Texas for a specific 2-year moving average period were then divided by the corresponding 2-year moving average of the total number of DKA hospitalization in our cohort. The result represented an approximated estimate of the fraction DKA hospitalizations of children and adolescents due to incident diabetes. This value allowed in turn the derivation of the remaining fraction of DKA hospitalizations, representing those due to prevalent diabetes in the state.

We examined the robustness of our approximation approach in 2 ways:

1. Because the reported data on the prevalence of DKA at time of diagnosis of type 2 diabetes were as high as 29% (3), we have recalculated the estimates of the fraction DKA hospitalizations due to incident diabetes, using a 30% prevalence rate of DKA at time of diagnosis of type 2 diabetes for the years 2005-2006, 2007-2008, 2008-2009 and 2009-2010.
2. We have estimated the prevalence of DKA at the time of diagnosis of type 1 diabetes required to have increase the fraction of DKA hospitalizations due to incident diabetes to 50% during the 2-year moving average 2010-2011 and 2011-2012 (the latest years with available incidence data (1)), while keeping the reported prevalence figures for the prevalence of DKA at diagnosis of type 2 diabetes during the years 2008-2010 (2) unchanged, rather than decreased (the prevalence of DKA at the time of diagnosis of type 2 diabetes mellitus reported by Dabalea et al for the years 2002-2003, 2004-2005, and 2008-2010 was 11.75, 6.3%, and 5.7% [p 0.005 for trend]).

The highest fraction of DKA hospitalizations due to incident diabetes, assuming the prevalence of DKA at the time of diagnosis of type 2 diabetes was 30%, ranged from 24.6% to 27.8%.

In order to have 50% of DKA hospitalizations due to incident diabetes during the years 2010-2011 and 2011-2012, the prevalence of DKA at the time of diagnosis of type 1 diabetes had to increase from 31.1% in 2009-2010 to 79.6% and 79.2% in 2010-2011 and 2011-2012, both highly implausible. Table S1 details the data used to approximate the fraction of DKA hospitalizations due to incident diabetes mellitus.

| **Table S1. Approximation of the fraction of pediatric DKA hospitalizations in Texas due**  **to incident diabetes** | | | | | | | | | | | | |  | |  | |  | | |  | |  |  |  |  |  |  |  |  |
| --- | --- | --- | --- | --- | --- | --- | --- | --- | --- | --- | --- | --- | --- | --- | --- | --- | --- | --- | --- | --- | --- | --- | --- | --- | --- | --- | --- | --- | --- |
| Year^a^ | Texas population | Texas population | | Incidence | Incidence | | Incident | | Incident | | DKA rate | | DKA rate | | Incident | | Total DKA | | |  | |  |  |  |  |  |  |  |  |
|  | 0-19 years^a^ | 10-19 years^a^ | | of T1DM^a,b,c^ | of T2DM^a,b,d^ | | T1DM^a,c,e^ | | T2DM^a,d,f^ | | T1DM^a,c,g^ | | T2DM^a,d,h^ | | DKA hospitalizations^a,i^ | | hospitalizations^a^ | | |  | |  |  |  |  |  |  |  |  |
| 2006 | 7,016,796 | 3,513,608 | | 20.8 | 8.2 | | 1,460 | | 288 | | 29.1 | | 6.3 | | 443 (21.3) | | 2,077 | | |  | |  |  |  |  |  |  |  |  |
| 2007 | 7,135,357 | 3,554,245 | | 21.4 | 9.0 | |  | |  | | NA^j^ | | NA | |  | |  | | |  | |  |  |  |  |  |  |  |  |
| 2008 | 7,211,658 | 3,568,934 | | 22.0 | 10.0 | | 1,587 | | 357 | | 31.1 | | 5.7 | | 513 (23.9) | | 2,149 | | |  | |  |  |  |  |  |  |  |  |
| 2009 | 7,291,125 | 3,582,225 | | 22.0 | 11.1 | | 1,604 | | 394 | | 31.1 | | 5.7 | | 522 (23.5) | | 2,219 | | |  | |  |  |  |  |  |  |  |  |
| 2010 | 7,476,856 | 3,677,009 | | 20.4 | 12.1 | | 1,525 | | 445 | | 31.1 | | 5.7 | | 499 (21.0) | | 2,383 | | |  | |  |  |  |  |  |  |  |  |
| a each period represents a 2-year average; thus, for example, the year 2006 represents a 2-year averaged (2005-2006) data for the specific domain | | | | | | | | | | | | | | |  | |  | | |  | |  |  |  |  |  |  |  |  |
| b Incidence of T1DM (or T2DM) represents the incidence of diabetes type, expressed per 100,000 population; based on data from reference 1 | | | | | | | | | | | | | | |  | |  | | |  | |  |  |  |  |  |  |  |  |
| c T1DM : type 1 diabetes | | |  | | |  | |  | |  | |  | |  | |  |  | |  | | | |  | | |  |  |  |  |
| d T2DM: type 2 diabetes | |  | |  |  | |  | |  | |  | |  | |  | |  | | |  | |  |  |  |  |  |  |  |  |
| e Incident T1DM represents an estimate of the number of new diagnoses of type 1 diabetes in Texas, based on the reported incidence and the corresponding Texas population | | | | | | | | | | | | | | | | |  | | |  | |  |  |  |  |  |  |  |  |
| example: the estimated number of children and adolescents newly diagnosed with type 1 diabetes during the 2006 period is derived from the corresponding incidence of 20.8/100,000 in a population of 7,019,796. | | | | | | | | | | | | | | | | |  | | |  | |  |  |  |  |  |  |  |  |
| f Incident T2DM represents an estimate of new diagnoses of type 2 diabetes in Texas based on based on the reported incidence and the corresponding Texas population. Example: the estimated number of children and adolescents newly diagnosed with type 2 diabetes during the 2006 period is derived from the corresponding incidence of 8.2/100,000 in a population of 3,513,608. | | | | | | | | | | | | | | | | |  | | |  | |  |  |  |  |  |  |  |  |
| g DKA rate T1DM: the rate of diabetic ketoacidosis at time of diagnosis of type 1 diabetes; based on data reported in reference 2 | | | | | | | | | | | | | | | | |  | | |  | |  |  |  |  |  |  |  |  |
| h DKA rate T2DM: the rate of diabetic ketoacidosis at time of diagnosis of type 2 diabetes; based on data reported in reference 2 | | | | | | | | | | | | | | | | |  | | | | | | |  | | |  | |  |
| i Incident DKA hospitalizations: the total estimated number of hospitalizations with a primary diagnosis of diabetic ketoacidosis with a newly  diagnosed type 1 and type 2 diabetes (e.g., incident diabetes) during a specific period; the figures in parentheses represent the percentage of all  reported DKA hospitalizations during a specific time period that are estimated to be due to incident diabetes.  Example: during the year 2006 there were estimated 1,460 children and adolescents newly diagnosed with type 1 diabetes, of which 29.1%  were expected to have DKA at the time of diabetes diagnosis, resulting in 425 DKA hospitalizations (assuming conservatively that all DKA  events result in hospitalization); similarly, out of the 288 children and adolescents diagnosed with type 2 diabetes, 6.3% were expected to have  DKA at the time of dagnosis, resulting in 18 DKA hospitalizations.  Together (425+18 = 443) these 443 DKA hospitalizations due to incident diabetes represent 21.3% of all DKA hospitalizations (2,077);  this means that during the 2006 period, 78.7% of DKA hospitalizations were estimated to be due to prevalent diabetes.  j NA : not available. Direct calcualtions could not be performed for this period because the SEARCH study data (reference 2) did not report the rate of diabetic keotacidosis for these years | | | | | | | | | | | | | | | | |  |  | | |  | | | |  | | |  |  |

**2) Results**

**Table S2. The annual volume of hospitalizations with diabetic ketoacidosis (DKA) and the corresponding Texas population, 2005-2014**

| **Categories** |  | **Year** | | | | |  | | | | |  |
| --- | --- | --- | --- | --- | --- | --- | --- | --- | --- | --- | --- | --- |
|  | 2005 | 2006 | 2007 | 2008 | 2009 | 2010 | | 2011 | 2012 | 2013 | 2014 | |
| **All Hospitalizations** | 1922 | 2231 | 2224 | 2074 | 2364 | 2401 | | 2718 | 2732 | 2615 | 2791 | |
| Population  **Age (years**) | 6560575 | 6708679 | 6778850 | 6853121 | 6932864 | 7241868 | | 7340045 | 7436085 | 7435862 | 7604179 | |
| 1month-<5 | 108 | 118 | 111 | 113 | 111 | 118 | | 158 | 153 | 146 | 155 | |
| Population | 1453964 | 1492885 | 1512285 | 1530851 | 1549067 | 1548627 | | 1548033 | 1558303 | 1537172 | 1568393 | |
| 5-<10 | 285 | 323 | 287 | 275 | 271 | 269 | | 313 | 307 | 313 | 340 | |
| Population | 1625457 | 1669732 | 1704137 | 1746831 | 1794787 | 1928234 | | 1942884 | 1960330 | 1956591 | 1987518 | |
| 10-<15 | 611 | 705 | 717 | 621 | 759 | 816 | | 890 | 904 | 863 | 825 | |
| Population | 1724651 | 1735753 | 1721492 | 1709339 | 1706081 | 1881883 | | 1932766 | 1954387 | 1993337 | 2053414 | |
| 15-19 | 918 | 1085 | 1109 | 1065 | 1223 | 1198 | | 1357 | 1368 | 1293 | 1471 | |
| Population | 1756503 | 1810309 | 1840936 | 1866100 | 1882929 | 1883124 | | 1916362 | 1963065 | 1948762 | 1994854 | |
| **Gender** |  |  |  |  |  |  | |  |  |  |  | |
| Male | 819 | 999 | 1044 | 917 | 1051 | 1119 | | 1262 | 1264 | 1197 | 1328 | |
| Population | 3355495 | 3430321 | 3465760 | 3503317 | 3543470 | 3706026 | | 3756719 | 3802576 | 3806742 | 3894076 | |
| Female | 1103 | 1232 | 1180 | 1157 | 1313 | 1282 | | 1456 | 1468 | 1418 | 1463 | |
| Population | 3205080 | 3278358 | 3313090 | 3349804 | 3389394 | 3535842 | | 3583326 | 3633509 | 3629120 | 3710103 | |
| **Race** |  |  |  |  |  |  | |  |  |  |  | |
| White | 916 | 1087 | 1124 | 965 | 1120 | 996 | | 1215 | 1197 | 1164 | 1210 | |
| Population | 2644306 | 2643136 | 2622727 | 2602061 | 2581391 | 2485573 | | 2470228 | 2505460 | 2440064 | 2468513 | |
| Hispanic | 493 | 554 | 583 | 580 | 668 | 786 | | 776 | 813 | 809 | 846 | |
| Population | 2860087 | 2958919 | 3039897 | 3124576 | 3215963 | 3458653 | | 3546174 | 3594676 | 3642764 | 3748425 | |
| Black | 379 | 439 | 383 | 388 | 440 | 483 | | 516 | 472 | 469 | 569 | |
| Population | 826776 | 867244 | 867831 | 869035 | 869378 | 867922 | | 870991 | 873747 | 869744 | 880345 | |
| Other | 131 | 150 | 133 | 139 | 136 | 136 | | 209 | 249 | 167 | 166 | |
| Population | 229406 | 239380 | 248395 | 257449 | 266132 | 429720 | | 452652 | 462202 | 483290 | 506896 | |

**Table S3. Changes in the number of failing organs and Deyo comorbidity index among hospitalizations with diabetic ketoacidosis in Texas**

|  | **Number of organ failures^a^** | |  | **Deyo comorbidity index^a^** | |  |  |
| --- | --- | --- | --- | --- | --- | --- | --- |
| **Category** | **2005-2006** | **2013-2014** | **p** | **2005-2006** | **2013-2014** | **p** |  |
| **All** | 0.09 (0.34) | 0.13 (0.40) | <0.0001 | 0.09 (0.34) | 0.14 (0.41) | <0.0001 |  |
| **Age (years)** |  |  |  |  |  |  |  |
| 1 months <5 | 0.08 (0.34) | 0.09 (0.34) | 0.7747 | 0.06 (0.23) | 0.10 (0.34) | 0.1629 |  |
| 5 - <10 | 0.06 (0.27) | 0.11 (0.32) | 0.2858 | 0.08 (0.29) | 0.11 (0.33) | 0.1421 |  |
| 10 - <15 | 0.07 (0.31) | 0.09 (0.34) | 0.0429 | 0.08 (0.33) | 0.13 (0.36) | 0.0001 |  |
| 15 - 19 | 0.11 (0.36) | 0.17 (0.44) | <0.0001 | 0.11 (0.36) | 0.15 (0.45) | 0.0020 |  |
| **Gender** |  |  |  |  |  |  |  |
| Female | 0.09 (0.33) | 0.11 (0.36) | 0.0731 | 0.09 (0.32) | 0.13 (0.41) | 0.0023 |  |
| Male | 0.09 (0.34) | 0.16 (0.44) | <0.0001 | 0.10 (0.36) | 0.14 (0.40) | <0.0001 |  |
| **Race/ethnicity** |  |  |  |  |  |  |  |
| white | 0.08 (0.32) | 0.13 (0.38) | <0.0001 | 0.09 (0.31) | 0.12 (0.39) | 0.0241 |  |
| Hispanic | 0.11 (0.36) | 0.11 (0.37) | 0.8112 | 0.08 (0.35) | 0.14 (0.42) | <0.0001 |  |
| black | 0.10 (0.36) | 0.19 (0.48) | <0.0001 | 0.11 (0.37) | 0.17 (0.43) | 0.0008 |  |
| other | 0.07 (0.28) | 0.12 (0.36) | 0.1121 | 0.10 (0.34) | 0.11 (0.35) | 0.7588 |  |
| **Health insurance** |  |  |  |  |  |  |  |
| Private | 0.09 (0.33) | 0.12 (0.38) | <0.0001 | 0.08 (0.31) | 0.12 (0.38) | 0.0026 |  |
| Medicaid | 0.09 (0.32) | 0.13 (0.41) | 0.0324 | 0.10 (0.36) | 0.16 (0.44) | <0.0001 |  |
| Self-pay | 0.10 (0.39) | 0.18 (0.44) | 0.0003 | 0.10 (0.38) | 0.12 (0.39) | 0.2929 |  |
| Other | 0.10 (0.30) | 0.12 (0.37) | 0.8187 | 0.16 (0.39) | 0.10 (0.32) | 0.1330 |  |
| ^a^ expressed as mean (standard deviation) | |  |  |  |  |  |  |
|  |  |  |  |  |  |  |  |
| **References** |  |  |  |  |  |  |  |

1. Mayer-Davis EJ, Lawrence JM, Dabelea D, Divers J, Isom S, Dolan L, Imperatore G, Linder B, Marcovina S, Pettitt DJ, Pihoker C, Saydah S, Wagenknecht L; SEARCH for Diabetes in Youth Study. [Incidence Trends of Type 1 and Type 2 Diabetes among Youths, 2002-2012.](https://www.ncbi.nlm.nih.gov/pubmed/28402773) N Engl J Med 2017;376:1419-1429.
2. Dabelea D, Rewers A, Stafford JM, Standiford DA, Lawrence JM, Saydah S, Imperatore G, D'Agostino RB Jr, Mayer-Davis EJ, Pihoker C; SEARCH for Diabetes in Youth Study Group. [Trends in the prevalence of ketoacidosis at diabetes diagnosis: the SEARCH for diabetes in youth study.](https://www.ncbi.nlm.nih.gov/pubmed/24685959) Pediatrics 2014;133:e938-45.
3. Scott CR, Smith JM, Cradock MM, Pihoker C. [Characteristics of youth-onset noninsulin-dependent diabetes mellitus and insulin-dependent diabetes mellitus at diagnosis.](https://www.ncbi.nlm.nih.gov/pubmed/9200365) Pediatrics. 1997 Jul;100(1):84-91.
